# Resection of a Pulmonary Hamartoma That Transformed into Stage IVB Lung Cancer over a 4-Year Period: A Case Report

**DOI:** 10.70352/scrj.cr.25-0107

**Published:** 2025-07-11

**Authors:** Yoshitaka Fujii, Tatsuya Nishida

**Affiliations:** Department of Thoracic Surgery, Ishikiriseiki Hospital, Higashiosaka, Osaka, Japan

**Keywords:** pulmonary hamartoma, malignant transformation, surgical resection

## Abstract

**INTRODUCTION:**

Pulmonary hamartoma (PH) is the most frequent benign tumor of the lung; however, the induction of malignant tumors and malignant transformation has been reported.

**CASE PRESENTATION:**

The patient was a woman in her 80s.Two transbronchial biopsies were performed for a 20-mm nodule in the S^8^ segment of the right lung, which showed a growing trend, and both were diagnosed as PH. Subsequently, she discontinued her outpatient visits but returned 4 years later with a complaint of blood in her sputum, and the right lung tumor had increased to 66 mm. In addition, a 35-mm tumor was found in the left lung S^1+2^ segment and a 35-mm tumor in the liver, and a diagnosis of cT3N0M1c stage IVB combined with large-cell neuroendocrine carcinoma was made. After chemotherapy, all tumors had shrunk, and no new lesions were detected, so the disease was judged to be oligometastatic, with localized metastases. Therefore, the patient underwent surgical resection of the primary tumor and radiotherapy for the metastases. As a result, the patient was alive and recurrence-free 8 months postoperatively.

**CONCLUSIONS:**

The possibility of accidental malignant transformation of components or surrounding tissues cannot be ruled out in PH, and careful follow-up and aggressive surgical resection should be considered, especially for lesions that increase in size over a short period.

## Abbreviations

ADL
: activities of daily living
CEA
: carcinoembryonic antigen
EBUS
: endobronchial ultrasonography
FDG-PET
: ^18^F-fluorodeoxyglucose positron emission tomography
LCNEC
: large-cell neuroendocrine carcinoma
OMD
: oligometastatic disease
PD-L1
: programmed cell death 1-ligand 1
PH
: pulmonary hamartoma

## INTRODUCTION

PH is a localized, mixed, abnormal tumor composed of normal bronchial components, such as the lineal epithelium, mucous glands, fat, muscle, and cartilage, and was 1st described as a tumor-like malformation by Albrecht in 1904.^1)^ According to the recent World Health Organization (WHO) classification, it is classified as a mesenchymal tumor specific to the lung^2)^ and is the most frequent benign tumor of the lung.

In general, PH is considered a benign tumor with a slow course, but malignant transformation has been reported.^3–5)^ The present case developed stage IVB lung cancer after a 4-year course despite being diagnosed with PH after 2 transbronchial biopsies, both of which were performed. After chemotherapy, it was determined that the metastases were localized (OMD), and surgical resection of the primary tumor and radiotherapy of the metastases were performed. No recurrence was observed 8 months after surgery, and the patient was doing well with no decline in ADL. The clinical course and treatment strategy of this case have been reported.

## CASE PRESENTATION

A woman in her 80s with an abnormal chest shadow detected during a health checkup was referred to the Department of Respiratory Medicine at our hospital in June 2020. The patient had a history of surgery for gastric cancer 27 years previously and uterine cancer 17 years previously and had comorbidities, such as bronchial asthma, hypertension, and rheumatoid arthritis. She had no history of smoking.

**Fig. 1:**
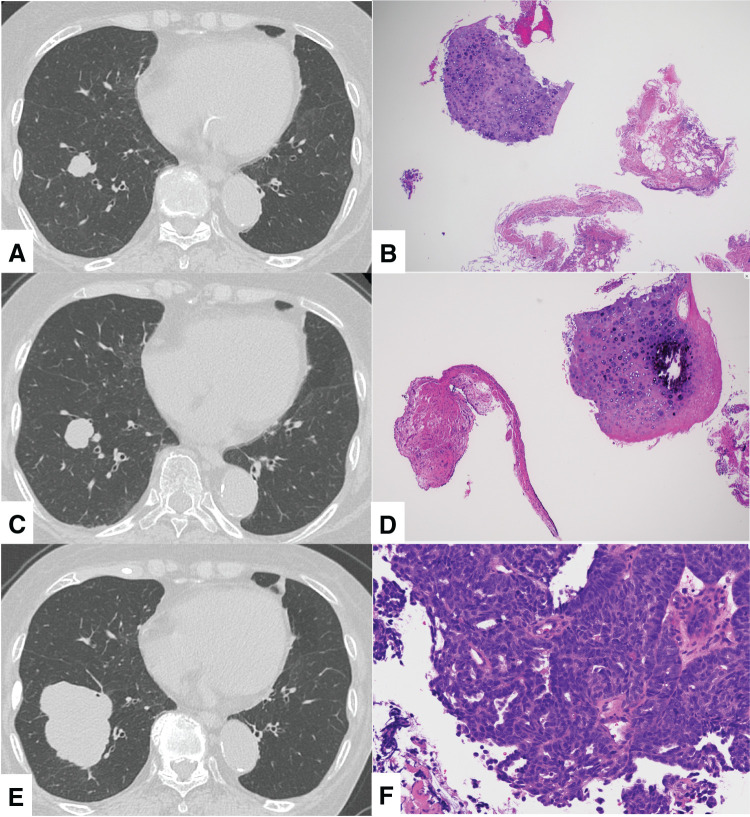
(**A**) Chest CT at the 1st examination (year 2020) showed a nodule measuring 17 mm in S^6^ of the right lower lobe. (**B**) A transbronchial biopsy revealed a large cartilage component on cytology, and the nodule was diagnosed as a hamartoma. (**C**) Chest CT taken 3 months after the 1st examination showed growth of the nodule to 23 mm in S^6^ of the right lower lobe. (**D**) A 2nd transbronchial biopsy revealed fibrous columnar epithelium and cartilaginous tissue on cytology, and the nodule was again diagnosed as a hamartoma. (**E**) Chest CT taken in year 2024 showed that the tumor in S^6^ of the right lower lobe had increased rapidly to 66 mm in size. (**F**) A transbronchial biopsy revealed hyperchromatic atypical cells proliferating in rosette, cord, or solid patterns, with some cribriform and papillary patterns (hematoxylin and eosin stain). Combined large-cell neuroendocrine carcinoma was suspected because CD56 and thyroid transcription factor 1 were positive, while chromogranin A, synaptophysin, cytokeratin 5/6, and p40 were negative on immunostaining.

Chest CT showed a 17-mm-diameter mass shadow in S^8^ of the right lung (**Fig. 1A**), and a transbronchial biopsy was performed using EBUS to ensure that a sufficient biopsy of the nodule was successfully performed, which revealed cartilage tissue and a diagnosis of PH (**Fig. 1B**). Although surgical resection was considered and the patient was referred to our department, she did not wish to undergo surgery and was followed up instead. On chest CT performed in September 2020, the nodule had grown to 23 mm in diameter (**Fig. 1C**), and a 2nd transbronchial biopsy was performed using EBUS to ensure that a sufficient biopsy of the nodule was successfully performed, which revealed fibrous columnar epithelium and cartilage tissue; the tumor was again diagnosed as PH (**Fig. 1D**). Although the tumor was benign, it had grown in a short period of time, and surgical resection was proposed again, but the patient did not wish to undergo surgery and continued to be followed up. Subsequently, the patient discontinued her outpatient visits.

**Fig. 2:**
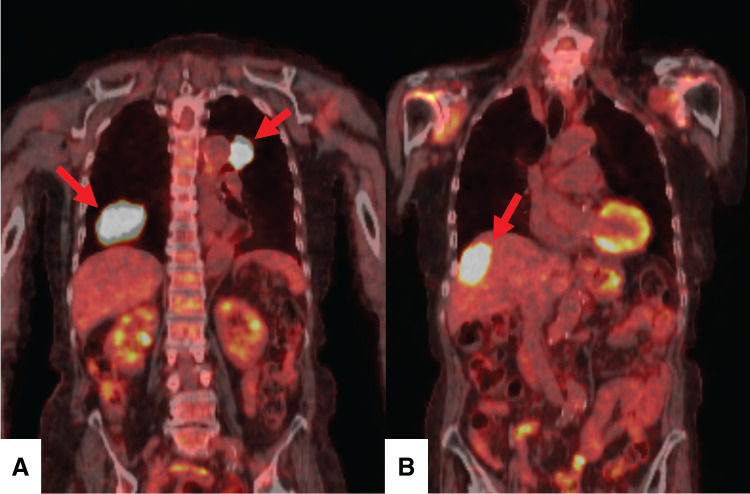
FDG-PET/CT in year 2024 showed the accumulation of FDG in (**A**) the right S^6^ and left S^1+2^ lung tumors (arrows) and (**B**) the liver tumor (arrow), but no accumulation in other areas. The patient was diagnosed with stage IVB lung cancer with multiple metastases in multiple organs. FDG-PET, ^18^F-fluorodeoxyglucose positron emission tomography

In December 2024, the patient visited another hospital complaining of blood in her sputum and returned to the Department of Respiratory Medicine because a chest radiograph showed a mass shadow in the right lower lung field and left pulmonary hilar region. Chest CT showed a mass shadow 66 mm in diameter in S^8^ of the right lung (**Fig. 1E**) and a mass shadow 35 mm in diameter in S^1+2^ of the left lung. Transbronchial biopsies were performed on both lung lesions, and both were diagnosed as combined LCNEC with adenocarcinoma components (combined LCNEC) (**Fig. 1F**). Contrast-enhanced magnetic resonance imaging of the head showed no brain metastases, but FDG-PET/CT showed a 35-mm liver tumor with significant FDG accumulation apart from the lung tumors (**Fig. 2**), suggesting liver metastases. Based on the above, lung cancer was diagnosed as cT3N0M1c stage ⅣB (8th edition).

The tumor was negative for genetic mutations and showed 0% expression of PD-L1. Based on this, a respiratory physician administered 4 courses of chemotherapy using carboplatin and nab-paclitaxel. The tumor shrank to 35 mm in the right lung, 21 mm in the left lung, and 25 mm in the liver, and no new lesions were observed. Therefore, although the patient had stage IVB right lung cancer, the cancer was determined to be OMD, and surgical resection of the primary tumor and radiotherapy of metastases were planned.

**Fig. 3:**
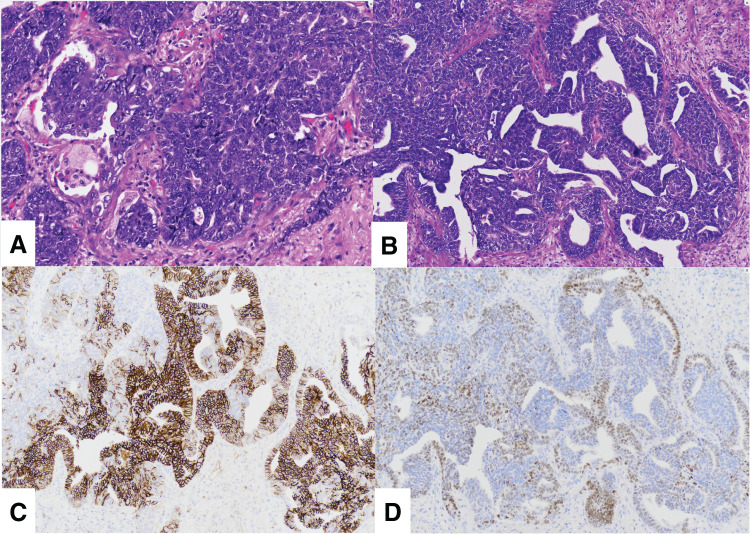
(**A**) Histopathological findings on excised specimens showed that hyperchromatic atypical cells proliferate in rosette, cord, or solid patterns. (**B**) Hyperchromatic atypical cells with cribriform, papillary, or micropapillary proliferation were observed in some areas. Immunostaining showed that the tumor cells were negative for chromogranin A and synaptophysin, and positive for (**C**) CD56 and (**D**) thyroid transcription factor 1. In addition, since cytokeratin 7 was positive and cytokeratin 20 was negative in the same area where CD56 was negative, a diagnosis of combined LCNEC was made. Furthermore, the absence of a hamartoma component led to the determination of malignant transformation from hamartoma to combined LCNEC. LCNEC, large-cell neuroendocrine carcinoma

After radiotherapy (total 48 Gy) for the left lung tumor, the patient was referred to our department for surgical resection of the tumor. On admission, the patient's height was 1.49 m, weight was 48.8 kg, and performance status was 0. Blood tests showed chemotherapy-induced leukemia and anemia, and elevated CEA levels of tumor markers (**Table 1**). Right lower lobectomy and hilar lymph node dissection were performed using a 4-port thoracoscopic approach. No obvious adhesions or disseminated lesions were observed in the thoracic cavity intraoperatively. A pathological examination of the excised specimen showed a mixture of LCNEC-like areas with chromatin-rich atypical cells proliferating in rosette, cord, and solid patterns (**Fig. 3A**), as well as adenocarcinoma components proliferating with cribriform, papillary, and micropapillary structures (**Fig. 3B**). Immunostaining was negative for chromogranin A and synaptophysin but positive for CD56 and thyroid transcription factor 1 (**Fig. 3C, 3D**), whereas in areas negative for CD56, cytokeratin 7 was positive and cytokeratin 20 was negative. Based on these findings, the patient was diagnosed with combined LCNEC. Furthermore, although a cartilage component was observed, the observed cartilage component was difficult to distinguish from bronchial cartilage, and there was no other component of PH in the tumor. As a result, this case was judged to be a malignant transformation of PH into a combined LCNEC.

**Table 1:** Laboratory data

	Initial examination	Before chemotherapy	At the time of hospitalization
WBC (/μL)	7550	10580	3260
RBC (×10^4^/μL)	414	398	227
Hb (g/dL)	12.9	12.7	8.1
Plt (×10^4^/μL)	27.1	31.6	12.6
CRP (mg/dL)	0.42	0.44	0.24
TP (g/dL)	7.0	6.6	6.2
T-bil (g/dL)	0.7	0.7	0.9
AST (IU/L)	20	21	19
ALT (IU/L)	22	19	18
LDH (IU/L)	316	232	263
γ-GTP (IU/L)	18	24	27
BUN (mg/dL)	17.0	16.3	13.0
Cre (mg/dL)	0.77	0.60	0.58
Na (mEq/L)	140	140	147
K (mEq/L)	4.4	4.0	3.7
Cl (mEq/L)	105	104	110
CEA (ng/ml)	3.7	8.0	15.5
CYFRA (ng/mL)	1.3	6.1	2.3
ProGRP (pg/mL)	24.1	34.9	21.9

ALT, alanine transaminase; AST, aspartate transaminase; BUN, blood urea nitrogen; CEA, carcinoembryonic antigen; Cre, creatinine; CRP, C-reactive protein; CYFRA, cytokeratin 19 fragment; γ-GTP, gamma-glutamyl transferase; Hb, hemoglobin; LDH, lactate dehydrogenase; Plt, platelet; ProGRP, progastrin-releasing peptide; RBC, red blood cell; T-bil, total bilirubin; TP, total protein; WBC, white blood cell

The postoperative course was uneventful, and the patient was discharged on postoperative day 9. After discharge from the hospital, radiotherapy was performed for the liver tumor (total 50 Gy), and at present, 8 months after surgery, the patient remains recurrence-free and has survived without additional chemotherapy, with no decline in ADL.

## DISCUSSION

The incidence of PH is 0.025%–0.040%, most commonly occurring in patients aged 50–60 years old, and the male-to-female ratio is reported to be 2–3:1.^6,7)^ It is generally asymptomatic and often incidentally detected on chest radiography as a peripheral lesion measuring 2–4 cm.^8)^ Although it is also characterized by well-defined popcorn-like calcification and fatty tissue,^9)^ it is not present in all cases, and it is difficult to diagnose based on imaging findings alone. The present case was an elderly patient with no calcification or fatty tissue, but was characterized by a small tumor diameter of 17 mm and well-defined borders at the time of initial examination.

Although PH grows, the rate of growth is slow, with an annual growth rate of 3.2 ± 2.6 mm^10)^ and a mean doubling rate of 36.4 ± 27.7 months.^11)^ The doubling rate of the present case was calculated using the method of Collins et al.^12)^ to be 75 days, which is extremely rapid and comparable to the doubling rates of lung malignancies (adenocarcinoma, 332 days; squamous cell carcinoma, 70 days; small-cell carcinoma, 51 days; and large-cell carcinoma, 192 days).^13)^ Therefore, the likelihood that the cartilage component collected in the transbronchial biopsy was incidental and that the present case may have had a malignant tumor from the time of the initial examination cannot be ruled out. However, it is difficult to differentiate between benign and malignant tumors based on the doubling rate alone, as there have been reported cases of PH in which the maximum diameter increased by 5 mm in 5 months.^14)^

There are various reports on the relationship between PH and lung malignancy, with the coexistence rate of PH and lung cancer being 5.0%–7.7%, and the incidence of lung cancer in PH is 6.3–6.6 times higher than that in cases without PH.^8,15)^ There have been reports of malignant transformation of PH into adenocarcinoma,^3)^ squamous cell carcinoma,^4)^ and sarcoma,^5)^ but no cases of transformation into LCNEC have been reported. Genetic abnormalities have been reported in PH,^16)^ and although it is conceivable that malignant tumors may arise from PH itself, it has also been pointed out that the presence of PH itself may stimulate the development of lung cancer.^17)^ In the present case, the possibility that a malignant tumor developed around the PH could not be ruled out, and it is possible that the enlargement of the PH may have contributed to the development of the malignant tumor. However, there is no clear scientific evidence, and further investigation is required.

Because PH is a benign tumor, enucleation or partial resection is generally chosen as the surgical procedure to preserve the lung.^18)^ However, some reports recommend segmentectomy or lobectomy because pulmonary malignancies tend to occur in the same lung lobe as PH.^15)^ Nevertheless, it is unreasonable to perform extensive resection without sufficient scientific evidence. Therefore, it is advisable to perform reduction surgery 1st for PH suspected of being malignant and then consider 1- or 2-phase expansive surgery depending on the results of the pathological diagnosis.

Regarding drug therapy for LCNEC, there are few studies with a high level of evidence, and the current treatment is similar to that used for small-cell carcinoma. In 1995, Hellman and Weichselbaum^19)^ proposed the concept of OMD, and local treatment, including surgery, was considered even for stage IV lung cancer if metastases were localized. OMD is a group of diseases encompassing a diverse range of conditions and no unified concept exists; however, in 2019, the European Organisation for Research and Treatment of Cancer published a consensus report, defining synchronous OMD as “five or fewer metastases within three organs, where all lesions can be treated locally”.^20)^ Furthermore, in 2020, the concept of subdividing OMD based on time horizon and treatment was proposed,^21)^ and the efficacy of local treatment, especially for synchronous OMD, has been investigated in several randomized trials, showing a prolonged progression-free survival or overall survival.^22,23)^ Because the present patient had combined LCNEC, was in her 80s, and did not express PD-L1, chemotherapy with carboplatin and nab-paclitaxel was administered instead of the usual therapy for small-cell carcinoma. As a result, the tumor shrank, but the CEA level did not decrease, and it was considered that the disease could not be adequately controlled by pharmacological therapy alone; therefore, surgical resection of the primary tumor and radiotherapy of the metastases were performed, as the present case met the definition of synchronous OMD. In the absence of clear evidence and considering the patient's advanced age, no adjuvant chemotherapy was administered; however, the patient was alive and recurrence-free at 8 months postoperatively, with no decline in ADL.

PH is often observed as a slowly growing benign tumor; however, considering the possibility of malignant transformation, early surgical resection should be actively considered in cases with a rapid doubling rate.

## CONCLUSIONS

Even in cases where a histological diagnosis of PH has been made, the possibility of malignant transformation must be considered if the tumor grows within a short period. Therefore, careful follow-up and aggressive surgical resection should be considered.

## DECLARATIONS

### Funding

The authors received no financial support for the preparation of this case report.

### Authors’ contributions

YF contributed to the study conception, data collection, and writing.

TN contributed to critical review and revision.

All authors read and approved the final manuscript.

### Availability of data and materials

Not applicable.

### Ethics approval and consent to participate

The patient provided written consent to undergo the procedures described in the case report. This case report did not require approval from our Institutional Ethics Committee.

### Consent for publication

The patient has permitted us to publish this case report.

### Competing interests

The authors declare that they have no conflicts of interest.
